# Healing after Trauma—New Knowledge and Procedures for the Benefit of Our Patients

**DOI:** 10.3390/life12050611

**Published:** 2022-04-19

**Authors:** Claudia Neunaber, Milena Fini, Paolo Cinelli

**Affiliations:** 1 Trauma Surgery Department, Experimental Trauma Surgery—Laboratory for Musculoskeletal Trauma and Regenerative Therapies, Hannover Medical School (MHH), Carl-Neuberg-Str.1, 30625 Hannover, Germany; 2 IRCCS Istituto Ortopedico Rizzoli, Complex Structure Surgical and Technological Sciences, Via di Barbiano, 1/10, 40136 Bologna, Italy; milena.fini@ior.it; 3 Department of Trauma Surgery, Center for Clinical Research, University Hospital Zurich, University of Zurich, Sternwartstrasse 14, CH-8091 Zurich, Switzerland; paolo.cinelli@usz.ch

Severe trauma is still the leading cause of death and disability in the world [1]. These severely injured patients have a particularly high risk of developing complications, which can lead to multiple organ failure or sepsis [2]. Moreover, trauma and fractures in old people aged over 70 years is rapidly growing. In 2017, more than 25% of all severely injured patients were belonging to the geriatric polytrauma group and showed significant differences in their injury patterns and treatment strategies due to pre-existing diseases as well as the intake of various drugs that alter the physiology [3]. Because the elderly population in the world continues to increase and elderly persons maintain independent and active lifestyles, this trend will increase further in the next years. Therefore, the underlying repair mechanisms after trauma must be elucidated intensively in order to be able to develop better therapeutic approaches for our patients and to face injury-related disabilities and complications. In this Special Issue, 12 publications provide new insights into novel treatments after serious injuries from basic science to clinical application with the following topics.

The publication of Lu et al. shows that a direct and current electrical field improves the wound healing ability in an experimental setting with human epidermal cells [4]. The migration and proliferation of cells exposed to electrical fields increased significantly compared to control group. The most abundant factor secreted by the cells following this procedure was granulocyte-macrophage colony-stimulating factor, which mediated its effect at least partially by activation of the Erk1/2 signaling pathway.

Feddahi et al. questions in an in vitro study if the needle design affects the regenerative potential of bone marrow aspirates, which are a promising alternative for the treatment of bone defects [5]. The authors harvested bone marrow from the posterior iliac crest of 12 patients either with Jamshidi needles or on the contralateral side with Marrow Cellution^®^ Needles. No significant difference could be found between the two different needle systems. Both acquired a sufficient and comparable number of about 40,000 mesenchymal stromal cells in 10 mL bone marrow aspirate.

The ideal time for iron administration in anemia secondary to blood loss was determined by Tiglis et al. through a 15% blood volume loss followed by a femur fracture stabilized using Kirschner wires in a rat model [6]. Intravenous ferric carboxymaltose infusion was administered 7 days and 48 h before surgery, intraoperatively, or 48 h after surgery. The control group was left anemic. The results indicated that correction of iron deficiency in emergency situations intraoperatively or 48 h postoperatively resulted in improved postoperative wound and bone healing compared to the anemic group or subjects who received a therapeutic intravenous infusion 48 h prior to surgery.

Furthermore, two publications deal with the inflammatory response after multiple traumas. In a retrospective study, Chen et al. evaluated the effect of fat distribution (visceral adipose tissue vs. subcutaneous adipose tissue) on the development of systemic inflammatory response syndrome in multiple trauma patients [7]. The results showed that lower visceral adipose tissue is associated with increased inflammatory response and worse clinical outcome in patients with polytrauma.

The group of Homeier et al. investigated the influence of the artificial IL-6 trans-signaling inhibitor sgp130Fc on posttraumatic cytokine levels to prevent a multi organ dysfunction syndrome caused by a dysbalanced posttraumatic immune reaction in a murine model consisting of femoral fracture and bilateral blunt chest trauma [8]. However, the results showed comparable cytokine levels between the treatment and control groups indicating neither a protective nor an adverse effect of sgp130Fc on the cytokine release after femoral fracture and bilateral chest trauma.

The influence of major surgical trauma on the function of natural killer cells and monocytes was determined by Müller-Heck et al. [9]. In this study, the impact of invasive spine surgery on the relationship between monocytes and NK cells upon exposure to *Staphylococcus aureus* was investigated up to eight days after surgery. Hereby, it was observed that NK cells continuously lost their ability to produce IFN-γ during the first week after surgery independently from monocyte-derived IL-12 secretion. Thus, NK cell suppression after major surgery might represent a therapeutic target to improve the immune defense against opportunistic infections.

The publication of Wagner et al. deals with the inhibition of pathologically increased matrix metalloproteinase (MMP) activity for the improvement of bone regeneration in mice with diabetes type 2 (db^−^/db^−^) compared to wildtype mice [10]. For this, a single dose of the broad-spectrum molecular MMP inhibitor Marimastat was locally applied into db^−^/db^−^ mice suffering from tibial bone defects. The application of Marimastat restored significantly impaired bone healing, collagen content, angiogenesis and osteoclast invasion in these mice compared to PBS. Hence, local intervention of bone defects by the molecular MMP inhibitor Marimastat might be an alternative therapeutic intervention for bone healing in diabetes.

Due to the incidence and increasing number of proximal humerus fractures, Oldrini et al. performed a systematic review and meta-analysis to evaluate the complications in patients treated with the internal fixation PHILOS plate system [11]. A total of 78 articles have been selected from 2004 to 2021. Complications were reported in 77 studies. Neer classification, reintervention rate, delto-pectoral and delto-split approach, patient age (>55 years), and functional outcome have been analysed. A total complication rate of 23.8% and a 10.5% reintervention rate increasing to 29.5% and 19%, respectively, in the over-55 population is reported. The most frequent complications are screw perforation (4.1% and 25.7% of all complications), humeral head avascular necrosis (3.1% and 17.9% of all complications), and subacromial impingement (1.5% and 10.1% of all complications). The authors concluded that there is the need of selecting types of patients and high-risk fractures to evaluate other solutions in patients affected by proximal humerus fracture.

In the publication of Brandes et al., a biomechanical study was performed to compare for the first time the Elastic Stable Intramedullary Nailing (ESIN) with a helical steel coil spring with limited bending flexibility (BoneHelix^®^, BH) for the surgical treatment of tibial shaft fractures in children and adolescents [12]. Porcine tibiae have been instrumented with the ESIN or BH device, and an unstable transversal bone fracture was created. Cyclic loading simulated a paediatric trauma with 18 weeks of postsurgical mobilization. CT examination demonstrated the absence of implant or bone failure with both treatments. None of the implants failed with a similar biomechanical performance by the two types of implants with higher stability for BH in the early weight bearing period paired with greater stiffness than ESIN.

Layher et al. tested the hypothesis that an internal fixator (IF) with a screw/rod system used in spinal surgery enables stable osteosynthesis of a femoral shaft fracture comparable to that achieved with an internal plate fixator (IP) [13]. The use of the IF could allow a one-stage definitive treatment of femoral fractures in severely injured patients. A non-displaced fracture has been created in artificial femurs and were treated with the two systems simulating different distal pin/rod distances and additional medial stabilization. The axial load distance phase during walking was simulated with biomechanical tests. The results showed that none of the unilateral IF combinations provided fracture stabilisation comparable to that of the IP specimen. Only by attaching a further rod, using just two additional screws inserted medially, the reference stiffness level and a comparable deformation were achieved.

Marchiori et al. proposed a multi-parametric approach on the relationship between joint function and soft tissue status before and after Anterior Cruciate Ligament (ACL) reconstruction by a graft [14]. A total of 13 consecutive patients were enrolled and joint laxity assessment and MRI with T2 mapping was performed in the pre-operative stage, at 4 and 18 months after surgery to acquire objective information correlating knee function and soft tissue condition. Correlations were found between graft and cartilage T2 signal, suggesting an interplay between these tissues within the knee joint. Graft maturation resulted connected to joint laxity. The presented integrated framework underlines the possibility to quantitatively assess the impact of ACL reconstruction on trauma recovery and cartilage homeostasis. The reported findings showed the possibility of monitoring the surgery outcomes using a multi-parametric prognostic investigation tool.

The scoping review of Di Sarsina et al. was aimed at analysing the clinical outcomes and failure rates of fresh osteochondral allograft transplantations (FOCA) in patients affected by Osteochondritis Dissecans (OCD) [15]. OCD affects a wide spectrum of patients, but it is most prevalent in adolescents and young adults. One of the current hypotheses for the origin of OCD is repetitive microtrauma. Reconstructive techniques for OCD of the knee are typically necessary when either non-operative or reparative/regenerative operative treatments fail, or the OCD is unsalvageable. Six papers have been selected from 1994 to 2018. Satisfactory clinical results and survival rates of the reconstruction with FOCA have been reported in the treatment of medium and large OCD lesions. The age at surgery and size of the lesion could affect graft survival, but there is insufficient data to state this definitively. Only high-quality comparative studies with other techniques could define the possible and real advantages of FOCA in the healing process of OCD lesions.

To conclude, we would like to thank all authors for sharing their knowledge in this Special Issue. We wish all readers a pleasant read that can be helpful by adding useful information and expanding readers’ knowledge. We hope that these new insights will contribute to the promotion and development of new and more effective treatments for severely injured patients that will improve quality of life and patient survival.
